# Motor Imagery and Action Observation: A Case for the Integration of 360°VR

**DOI:** 10.3389/fpsyg.2022.880185

**Published:** 2022-04-26

**Authors:** Riki Lindsay, Aden Kittel, Michael Spittle

**Affiliations:** College of Sport and Exercise Science, Institute for Health and Sport, Victoria University, Melbourne, VIC, Australia

**Keywords:** mental imagery, immersive video, sport psychology, virtual reality, neuroscience

## Introduction

There are several situations that can cause an athlete's regular training volume to be reduced, such as injury, traveling overseas for competition, or highly relevant to the current world climate, inability to attend a training venue due to infection risk (i.e., COVID-19). Subsequently, practitioners have sought supplementary training methods that can complement or be added to physical training to meet the needs of their athletes. One such training alternative is motor imagery (MI), defined as a simulation state that involves covertly rehearsing a motor action mentally by incorporating visual and kinesthetic aspects of movement, without actually executing movement (Frank et al., 2014; Eaves et al., 2016). MI is most effective when combined with physical training, providing an additive effective to performance (Lindsay et al., 2021). Considerable evidence exists attesting to the beneficial effects of MI for performance and skill development (e.g., Simonsmeier et al., 2020; Lindsay et al., 2021). Motor Simulation Theory (MST) proposes that the positive effects of MI can in part be attributed to shared neural mechanisms with physical practice, termed *functional equivalence* (Jeannerod, 1994, 1995; Debarnot et al., 2014; Moran and O'Shea, 2020). Consistent with MST, Hétu et al. (2013) found that MI activated areas of the brain similar to that observed during motor execution including premotor and parietal cortices, and fronto-parietal regions. Further evidence indicates that MI may also be capable of producing similar training-related adaptations in central neural structures as physical training. For example, Leung et al. (2013) showed that increases in corticospinal excitability were equivalent following MI training relative to physical training of a bicep-curl, suggesting similar changes in motor execution processes.

More recently, researchers have advocated for combining techniques such as action observation (AO) with MI to facilitate greater improvements in performance relative to MI or AO delivered as separate interventions (Eaves et al., 2016; Wright et al., 2021). AO involves observing actions displayed via video or physical demonstration of another individual or self with the intention of replicating the observed movement (Wright et al., 2021). The effectiveness of AO for improving motor skill performance and learning has commonly been contextualized through the notion that observation of motor actions activates an action observation network (AON), comprising of similar motor areas of the brain (e.g., premotor cortex, inferior parietal lobule and supplementary motor area) utilized during physical action (Cross et al., 2009).

## Combining Action Observation and Motor Imagery

Previous research has often treated MI and AO as separate interventions (Jeunet et al., 2020), however, there is an emerging body of evidence to suggest that both techniques can be combined or structured so that AO precedes MI to act as a visual primer of the movement being practiced (Kim et al., 2017; Romano-Smith et al., 2018). Treated as a single technique, AOMI interventions have been shown to be more effective for enhancing learning and performance relative to AO or MI alone (Eaves et al., 2016). For example, Romano-Smith et al. (2018) found that participants practicing a manual aiming task with either simultaneous or alternate AOMI improved performance significantly more than MI or AO alone. The success of AOMI has largely been attributed increases in neural activity in motor regions of the brain, which exceeds levels produced in MI or AO alone (Romano-Smith et al., 2018). This is supported by neurophysiological research suggesting that AOMI produces more significant cortico-motor activity compared to AO alone (Wright et al., 2014). Wright et al. (2018) found that AOMI facilitated significantly greater corticospinal excitability relative to AO or MI alone when practicing a basketball free-throw. Overall, the emerging body of evidence provides some encouraging findings to suggest that AOMI could facilitate greater neurophysiological activity in motor-related brain areas, potentially contributing to improved motor skill and learning outcomes.

Though evidence indicates clear benefits of AOMI, one element that deserves further consideration is the type of video footage used during AOMI. Presently, AOMI interventions have primarily utilized 2-D video footage from the first-person perspective (Romano-Smith et al., 2018; Scott et al., 2018). An issue related to the type of video footage is its influence on the sense of agency (SoA) for the participant. SoA is described as an individual's experience of the level of control and initiation they have over an action (Braun et al., 2018). Further, SoA and the degree of immersion an individual experiences within virtual environments have been proposed to be important factors in the efficacy of virtual training (Rose et al., 2018). Immersion is described as the degree of which simulated environments produce experiences that accurately replicate the multimodal sensory nature of the real-world (Rose et al., 2018). Recent research suggests that the degree of immersion in virtual environments can influence an individual's perception of whether they really have control over their own movements (i.e., SoA; Kong et al., 2017). However, further research is needed to understand the relationship between immersion and SoA.

Generally, researchers conducting an AOMI intervention will ask participants to imagine themselves executing the motor skill; whereas, the video is usually a recording of another person performing the skill. According to the comparator model of motor control (Frith, 2005; David et al., 2008), the level of control of a movement is governed by an internal prediction model within the brain (David et al., 2008). When a new motor command is produced, this causes creation of an efference copy. If the efference copy is the same as the actual sensory input (e.g., AO video footage) the motor action is interpreted as being self-caused, leading to SoA (Braun et al., 2018). Captured this way, it is possible that using video footage during AO of another individual may result in a mismatch between the efference copy and the sensory input (AO of another individual), culminating in a lack of perceived self-caused movement (Braun et al., 2018). Previous MI research seems consistent with this idea, suggesting that kinesthetic MI may be inhibited when viewing another person (Callow and Hardy, 2004). In order to create a representative AOMI experience for the individual understanding how to create a SoA is an important consideration for the practitioner to ensure that the simulated movement is perceived as self-caused.

## Virtual Reality for Motor Imagery

Alternative technologies, such as virtual reality (VR), present a viable solution for improving SoA of AOMI interventions by generating a more interactive and immersive practice environment. Given the increased neurophysiological activity reported during AOMI, authors support this combined approach as being the optimal method for mental simulation interventions in sport (McNeil et al., 2021; Wright et al., 2021). For example, Im et al. (2016) reported that virtual-reality-guided MI of a wrist extension task increased cortical excitability in both stroke patients and healthy participants more than MI alone. Similarly, Bedir and Erhan (2021) reported significant improvements in shot accuracy in curling, bowling and archery athletes following VR-based imagery relative to Visual Motor Behavior Rehearsal and Video Modeling. In addition, it was noted that the VR component may have contributed to participants adapting to imagery training earlier due to the enhanced simulation of kinesthetic, visual and auditory senses. VR can improve the level of “presence,” which is defined as how much individuals feel immersed within an environment (Slater, 2018; Bird, 2020). VR typically presents virtual scenarios enabling the participant to interact (Düking et al., 2018). These VR scenarios, however, may limit ecological validity, which refers to similarity of perceptual information in the simulation to the real-world (i.e., competitive environment) (Araujo et al., 2007).

## 360°VR as an Alternative

**Figure 1:**
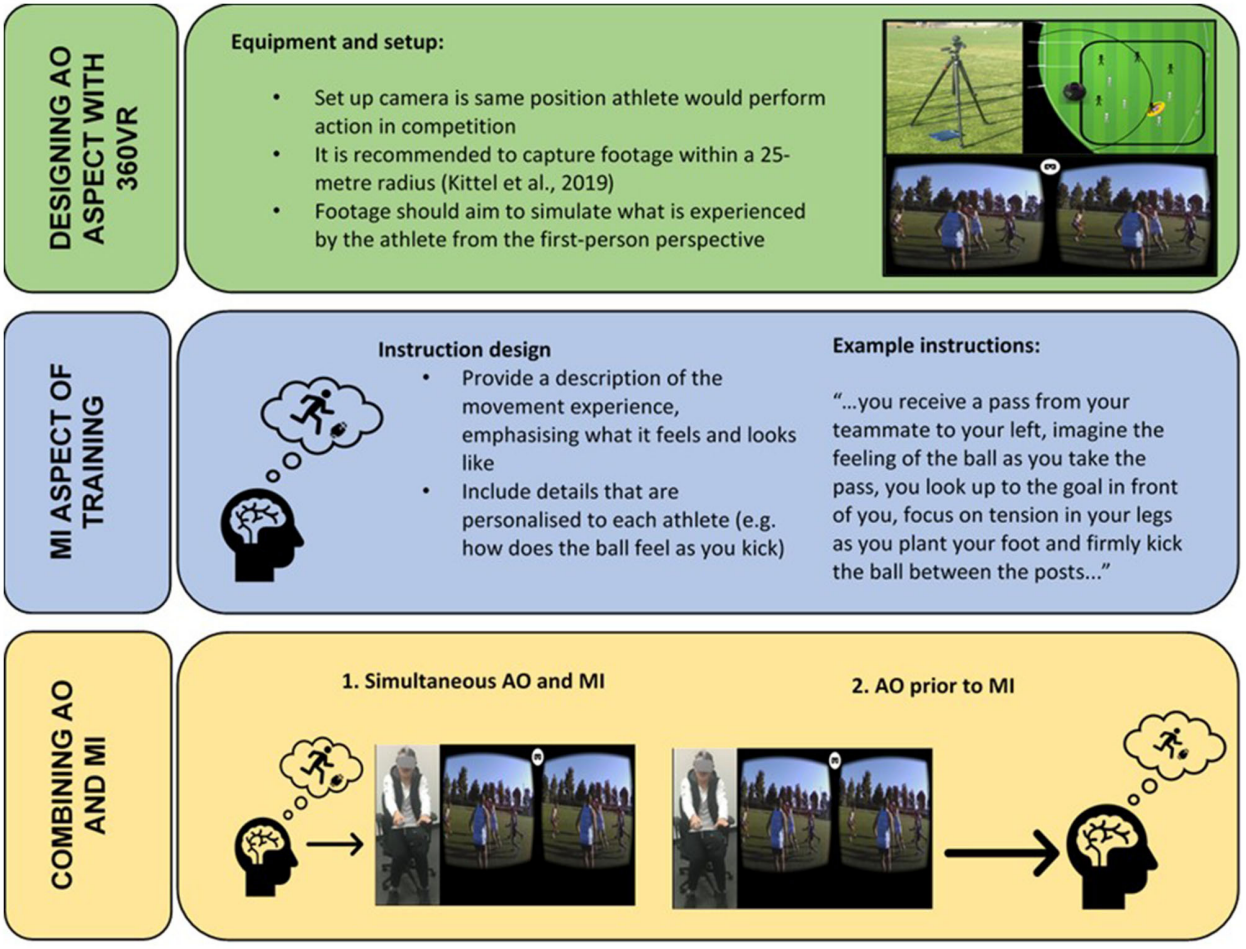
Example of how 360°VR and MI can be implemented for practicing skills in Australian Rules Football. Adapted from recommendations provided by Wright et al. (2021).

360°VR overcomes potential ecological validity limitations of VR, by presenting real-world immersive video, while also using a head-mounted display (HMD) similar to VR [see Kittel et al. (2020a) for a SWOT analysis of this technology]. Harris et al. (2020) explain that physical fidelity and realism are vital for the “VR equivalent of mental imagery” (p. 4), however, this suggests that VR and MI are always separate. We suggest that 360°VR deserves consideration as the AO component of combined AOMI training as it provides realistic simulations that can be created in an easy cost-effective manner. Realism is a vital component of presence (Schubert et al., 2001), suggesting the vision provided through 360°VR would lead to greater presence than virtual environments (i.e., VR). 360°VR presented using a HMD allows participants to scan the environment using head movements, which in turn increases the visual flow perceived (Craig, 2013; Pagé et al., 2019). Participants have the ability to attune to cues they perceive to be relevant, rather than flat-screen video providing a limited number of cues, given the less interactive nature of the presentation. This is pertinent when considering the importance of SoA. Flat screen video does not allow participants to scan, potentially diminishing the SoA in AOMI. Incorporating 360°VR as the AO component is likely to increase the perception of SoA. This would allow a more personalized and exploratory approach, as research has indicated that personalized MI can provide benefits over generic MI (Wilson et al., 2010). An important consideration is that individuals may not be able to generate and control MI as prescribed by the coach or sport psychologist (Wright et al., 2021). Therefore, 360°VR presents a viable addition to facilitate MI generation that could be the missing link for providing the initial vivid imagery required. Wright et al. (2021) explain that when using flat-screen video as the AO component, the practitioner can control the viewing perspective. However, 360°VR enables the performer to go one step further by allowing them the opportunity to scan through the environment, as they would in competition. Figure 1 provides an example of how 360°VR and MI could be implemented for practicing skills in Australian Rules Football adapted from recommendations provided by Wright et al. (2021).

When developing 360°VR, there are several technical considerations for presenting this innovative footage. For example, there are several different 360°VR cameras available at different costs. These can range from several hundred dollars (2022 retail price) such as the 360fly4k (single lens) used by Kittel et al. (2019) and the Ricoh Theta S 360° camera (dual lens) used by Pagé et al. (2019). There are options that are more expensive available such as six Go-Pros in a cube (Panchuk et al., 2018). There is a trade-off between better quality cameras that use multiple lenses, but require significantly more post-production stitching the footage together. Although HMDs present stereoscopic vision that allows the viewer to perceive the depth of objects and increase representativeness (Farley et al., 2020), the headsets can differentiate the quality of the video. For example, there are lower costing (and lower resolution) such as the Utopia 360 HMD (Pagé et al., 2019) to tethered HMDs with in-built screens that allows other individuals to see the 360°VR through an attached display (Oculus Rift; Panchuk et al., 2018). The Oculus Go may be an appropriate middle ground, given it is affordable and uses an in-built screen, but is lower in price and not tethered to a display. While VR may cause issues with latency between their perception and the virtual avatar (for a more technical overview of 360°VR, please see Farley et al., 2020), this is not an issue with 360°VR as the participant is not interacting with a virtual avatar. Future research is required to understand these technical elements of 360°VR, such as utilizing monoscopic versus stereoscopic views.

As highlighted earlier, presence is vital for simulated environments, whether that be MI or AO in a virtual environment. To be immersed and present, there needs to be a strong sense of plausibility, namely the feeling that the situation is actually occurring (Slater and Sanchez-Vives, 2016; Harris et al., 2020). Given the high levels of ecological validity that 360°VR provides (Kittel et al., 2019), this can offer increased plausibility for MI for the individual to feel the situation is real. A reported limitation of using 360°VR is the difficulty in combining perception and action, given participants are observing a real environment, rather than interacting within a virtual environment (Kittel et al., 2020a). Incorporating MI with 360°VR technology as the AO component could help mitigate this, as MI can include the kinesthetic (i.e., moving component). Theoretically, this may increase the perception-action coupling that is not always present when using 360°VR technology (Fadde and Zaichkowsky, 2018). 360°VR has demonstrated positive long-term behavioral changes in perceptual-cognitive skills such as decision-making (Kittel et al., 2020b), which could be attributed to greater embodiment, allowing greater sensorimotor engagement for behavioral adaptations (Bohil et al., 2011; Kilteni et al., 2012). The implementation of this technology as the AO component could enhance the optimized long-term memory changes gained with the combination of AOMI (Kim et al., 2017; Wright et al., 2021). From a practical standpoint, 360°VR presents an affordable option for practitioners to present AO in a more immersive way. 360°VR is significantly financially more affordable and accessible than VR (Düking et al., 2018; Kittel et al., 2020a). 360 cameras are relatively comparable in price to standard cameras, and when presented through a HMD, this is significantly less expensive than the associated costs of developing and presenting a virtual environment in VR. Importantly, 360°VR presents a more novel presentation method for AO, with this technology rated as more enjoyable and relevant as a perceptual-cognitive tool than screen-based video (Kittel et al., 2020b).

## Conclusion

In conclusion, 360°VR provides an innovative tool to be used in conjunction with MI in sport. Given the current climate, in which athletes may be spending long periods in quarantine situations before competitions, simulated training techniques such as AOMI may prove an invaluable technique in the athletes' toolbox. The enhanced realism of 360°VR lends a greater sense of presence and embodiment for the individual to be immersed in the imagined environment. Furthermore, the SoA is improved by the individual's ability to scan within a real-world environment. As a result, 360°VR allows MI to provide consistent simulations for athletes, with AO and MI combined for greater neurophysiological stimulation. With the long-term behavioral changes afforded by AOMI and 360°VR, the integration of this technology as the AO component would theoretically enhance SoA and should be investigated further from a theoretical and applied perspective in the literature.

## Author Contributions

RL, AK, and MS contributed to conception and structure of this article and writing the first draft of the manuscript. All authors were involved in manuscript revision, reading, and approval of the submitted version.

## Funding

This work was supported by the Institute for Health and Sport, Victoria University.

## Conflict of Interest

The authors declare that the research was conducted in the absence of any commercial or financial relationships that could be construed as a potential conflict of interest.

## Publisher's Note

All claims expressed in this article are solely those of the authors and do not necessarily represent those of their affiliated organizations, or those of the publisher, the editors and the reviewers. Any product that may be evaluated in this article, or claim that may be made by its manufacturer, is not guaranteed or endorsed by the publisher.
